# Predicting bacterial transport through saturated porous media using an automated machine learning model

**DOI:** 10.3389/fmicb.2023.1152059

**Published:** 2023-05-10

**Authors:** Fengxian Chen, Bin Zhou, Liqiong Yang, Xijuan Chen, Jie Zhuang

**Affiliations:** ^1^Key Laboratory of Pollution Ecology and Environmental Engineering, Institute of Applied Ecology, Chinese Academy of Sciences, Shenyang, Liaoning, China; ^2^Faculty of Medicine, University of Augsburg, Augsburg, Germany; ^3^Department of Biosystems Engineering and Soil Science, Center for Environmental Biotechnology, The University of Tennessee, Knoxville, TN, United States

**Keywords:** bacterial transport, automated machine learning, first-order attachment coefficient, spatial removal rate, machine learning

## Abstract

*Escherichia coli*, as an indicator of fecal contamination, can move from manure-amended soil to groundwater under rainfall or irrigation events. Predicting its vertical transport in the subsurface is essential for the development of engineering solutions to reduce the risk of microbiological contamination. In this study, we collected 377 datasets from 61 published papers addressing *E. coli* transport through saturated porous media and trained six types of machine learning algorithms to predict bacterial transport. Eight variables, including bacterial concentration, porous medium type, median grain size, ionic strength, pore water velocity, column length, saturated hydraulic conductivity, and organic matter content were used as input variables while the first-order attachment coefficient and spatial removal rate were set as target variables. The eight input variables have low correlations with the target variables, namely, they cannot predict target variables independently. However, using the predictive models, input variables can effectively predict the target variables. For scenarios with higher bacterial retention, such as smaller median grain size, the predictive models showed better performance. Among six types of machine learning algorithms, Gradient Boosting Machine and Extreme Gradient Boosting outperformed other algorithms. In most predictive models, pore water velocity, ionic strength, median grain size, and column length showed higher importance than other input variables. This study provided a valuable tool to evaluate the transport risk of *E.coli* in the subsurface under saturated water flow conditions. It also proved the feasibility of data-driven methods that could be used for predicting other contaminants’ transport in the environment.

## Highlights

- The predictive models showed better performance when bacterial retention was high.
- Spatial removal rate is a better target variable than first-order attachment coefficient.
- Algorithms based on gradient boosting outperformed other machine learning algorithms.

## 1. Introduction

Microbiologically contaminated drinking water is estimated to cause 485,000 diarrhoeal deaths each year (WHO, 2022). Manure-borne pathogens, as an important source of microbiological contamination, can be transported from surface soil to groundwater through manure disposal, storage, and land application, posing a threat to public health (Pachepsky et al., 2006; Alegbeleye and Santana, 2020). The vertical transport of bacteria in soil is an important route for microbiological contamination, especially in the area with shallow groundwater level (Guo et al., 2020). Numerous flow-through experiments, from lab-scale to field-scale, were conducted to identify the key parameters and explore the mechanism of bacterial transport through porous media or natural soils (Schinner et al., 2010; Bradford et al., 2013; Yang et al., 2019; Oudega et al., 2021; He et al., 2022). The results showed that the bacterial transport process was controlled by environmental factors (e.g., soil texture, particle size, soil surface charges, organic matter content, water content, ionic strength, water flow velocity) and bacterial properties (e.g., concentration, cell size, cell surface charge, hydrophobicity) (Sepehrnia et al., 2017; Zhong et al., 2017). Based on these experimental data, mathematical models, such as attachment/detachment model and filtration theory, were used to describe and compare the bacterial transport behaviors (Šimunek et al., 2012). These studies are rigorous enough to emphasize some specific factors of bacterial transport in each independent study. However, they have two obvious limitations. One is the difficulty to evaluate many variables at one time, specifically, as the number of variables increases, the workload of experiments will grow exponentially. Second, the model parameters from one specific experiment may be not suitable for predicting bacterial transport in other scenarios because of different soil properties, scales, and water flow conditions. Therefore, data-driven methods beyond the experiments are needed for the prediction of bacterial transport under a wide range of scenarios.

In recent years, data-driven methods have been developed fast due to the advances in machine learning (Solomatine et al., 2009; Hiemer and Zapperi, 2021). Machine learning is the process of generating models that learning from historic data to make predictions for the future or other scenarios (Samanpour et al., 2018). Previous bacterial flow-through experiments produced large amount of data, providing a foundation for developing data-driven models such as machine learning. However, the machine learning approach demands a high level of technical sophistication in model selection and hyperparameter tuning, constituting an obstacle for non-machine learning experts (Hutter et al., 2019). Thus, automated machine learning (AML) was proposed in recent years. The AML approach can automatically optimize machine learning process by integrating feature engineering, model selection, hyperparameter optimization, and model evaluation (Waring et al., 2020). The recent AML models include AutoWEKA (Kotthoff et al., 2019), Auto-sklearn (Feurer et al., 2020), AutoGluon (Erickson et al., 2020), H2O AutoML (LeDell and Poirier, 2020), and TPOT (Olson and Moore, 2016). They were used for analysis and prediction in many areas of environmental science, such as groundwater redox conditions, landslide hazard analysis, methane production in anaerobic digestion, and groundwater radioactivity (Wilson et al., 2020; Qi et al., 2021; Fallatah et al., 2022; Xu et al., 2022). However, they have not been applied to predict environmental fates of pathogens and pollutants.

The first step for AML modeling is to define the input variables (known as features) and target variables (Waring et al., 2020). For the vertical transport of bacteria in soil, the input variables can be bacterial properties and environmental factors, while the target variables should be parameters that can effectively describe the transport and retention behaviors of bacteria using the advection-dispersion equation as follows,


(1) $$\frac{\partial C}{\partial t}=D\frac{\partial^{2}C}{\partial x^{2}}-v\frac{\partial C}{\partial x}-kC$$

In the equation, C (cell mL^−1^) is the bacterial concentration, t (h) is time, x (cm) is travel distance, D is dispersion coefficient (cm^2^ h^−1^), v (cm h^−1^) is the pore water velocity and k (h^−1^) is first-order attachment coefficient (Kretzschmar et al., 1997). For a given scenario, the parameter C,t,x,D,v can be easily measured or calculated. If k can be predicted as a target variable, the bacterial transport and retention can be described. The simplified bacterial retention in soil can be written as


(2) $$\frac{dC}{dt}=-kC$$

This exponential decay function describes that the bacterial concentration decreases at a rate proportional to its current concentration value, and k is an exponential decay constant. Besides, the bacterial removal can also be described from distance perspective,


(3) $$\frac{dC}{dx}=-\lambda C$$

where λ (cm^−1^) is spatial removal rate of bacteria (Kretzschmar et al., 1997; Pang, 2009). The exponential decay function describes that the bacterial concentration decreases along the distance at a rate proportional to its current concentration value, and λ is an exponential decay constant.

Both parameters k and λ describe the bacterial transport and retention in soil. The former is time based, while the latter is distance based. The relation between k and λ under steady state flow is k=λv.

*Escherichia coli* (*E. coli*) is commonly used as a common indicator of fecal contamination (Odonkor and Ampofo, 2013). In this study, we extracted 377 datasets of *E. coli* vertical transport in saturated porous media from 61 papers. The objective of this study was to predict the two main parameters of *E. coli* transport in saturated porous media using an AML model. The input variables were bacterial concentration, porous medium type, median grain size, ionic strength, pore water velocity, column length, saturated hydraulic conductivity, and organic matter content, and the target variables were first-order attachment coefficient and spatial removal rate. Based on the AML model (H2O AutoML), six types of machine learning algorithms and 20 predictive models were trained, and their performances were evaluated.

## 2. Materials and methods

### 2.1. Data collection and analysis

A literature search was conducted on the Web of Science to collect data regarding the *E. coli* transport in soil/sand (Supplementary Table S1). Reducing the number of input variables is beneficial for improving the model prediction performance. From the collected literature, we selected eight most frequently investigated factors as input variables, including bacterial concentration, porous medium type, median grain size, ionic strength, pore water velocity, column length, saturated hydraulic conductivity, and organic matter content. Some bacterial properties, such as cell size (with a length of 1–2 μm and a radius of 0.5 μm) and hydrophobicity (mostly hydrophilic), were not considered because their difference among different *E. coli* strains was small. The bacterial zeta potential (−10 ~ −50 mV) was not considered because it was strongly correlated with the ionic strength of liquid phase. Among the eight input variables, porous medium type was categorical variable (i.e., sand, intact soil, and disturbed soil), and the other seven variables were numerical variables.

The target variable (the first-order attachment coefficient *k*) was collected through the following ways: (1) when the first-order attachment model (Equation 1) was used to fit break-through curves, k was an optimized parameter; (2) if the breakthrough curves were not fitted with models or not fitted by the first-order attachment model, k was converted from λ based on k=λv .

The target variable (spatial removal rate λ) was collected through the following two ways. First, in breakthrough curves, when the effluent concentration reached a plateau, λ was calculated from the following equation:


(4) $$\lambda =-\frac{1}{L}\ln\left(\frac{C_{f}}{C_{0}}\right)$$

where L is the length of the soil column, $C_{0}$ is the bacterial input concentration, and $C_{f}$ is the effluent concentration at the plateau of the breakthrough curve (Kretzschmar et al., 1997). Second, for the breakthrough curves without a plateau, λ was calculated using the following equation:


(5) $$\lambda =-\frac{1}{L}\ln\left(\frac{M_{\mathrm{eff}}}{M_{\mathrm{in}}}\right)$$

where is *M*_in_ the total injected bacterial mass and *M*_eff_ is the total effluent bacterial mass (Kretzschmar et al., 1997).

If *k* is easily obtained, λ is converted by k=λv . In the study, the values of *k* and λ was extracted from the literature or otherwise calculated from the breakthrough curves.

### 2.2. Automated machine learning model

**Figure 1:**
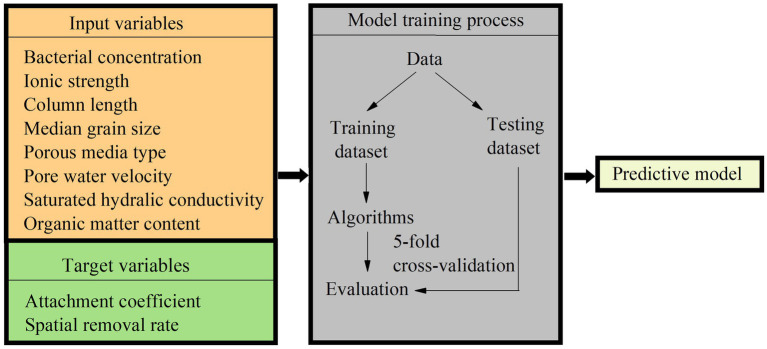
The variables and machine learning model training process in this study.

We used an automated machine learning model, H2O AutoML (LeDell and Poirier, 2020). The H2O AutoML integrated many common machine learning algorithms, including Deep Learning, Distributed Random Forest (DRF), Generalized Linear Model (GLM), Gradient Boosting Machine (GBM), Extremely Randomized Trees (XRT), and Extreme Gradient Boosting (XGBoost) (LeDell and Poirier, 2020). The H2O AutoML provides some function calls for automatically training the candidate models. The codes in R for modeling training and model performance evaluation were shown in the Supplementary material. The variables and machine learning model training process are shown in Figure 1.

### 2.3. Model performance measures

The collected bacterial transport datasets were randomly divided into two datasets: 80% training dataset and 20% test dataset. The training dataset was used to train the machine learning models and the test dataset was used to evaluate the model performance. 5-fold cross-validation was performed. Based on the test dataset and the model predicted target variables, three statistical measures were used to evaluate the model performance: root mean squared error (RMSE), mean absolute error (MAE) and absolute relative residual (ARR). For each predicted value, there is one ARR value (Li et al., 2020).


(6) $$\mathrm{RMSE}=\sqrt{\frac{1}{n}\overset{n}{\underset{i=1}{\sum }}{\left({\hat{y}}_{i}-y_{i}\right)}^{2}}$$

(7) $$\mathrm{MAE}=\frac{1}{n}\overset{n}{\underset{i=1}{\sum }}\left|{\hat{y}}_{i}-y_{i}\right|$$

(8) $$\mathrm{ARR}=\left|\frac{{\hat{y}}_{i}-y_{i}}{y_{i}}\right|$$

where ${\hat{y}}_{i}$ is the model predicted value, $y_{i}$ is the observed value, and n is the number of data points in the test dataset.

### 2.4. Explainable analysis

In H2O AutoML, 20 machine learning algorithms was synchronously examined, and their ranking was listed on a leaderboard based on their performance. Variable importance and Shapley additive explanations (SHAP) were used to analyze the importance and contribution of the input variables (LeDell and Poirier, 2020). The variable importance is ranged from 0 to 100% and represents the importance of each input variable for the target variable. SHAP shows the contribution of each variable in each row of data and the trend of the variable’s influence, i.e., positive, or negative influence (Lundberg and Lee, 2017).

## 3. Results and discussion

### 3.1. Overview of the collected datasets

**Figure 2:**
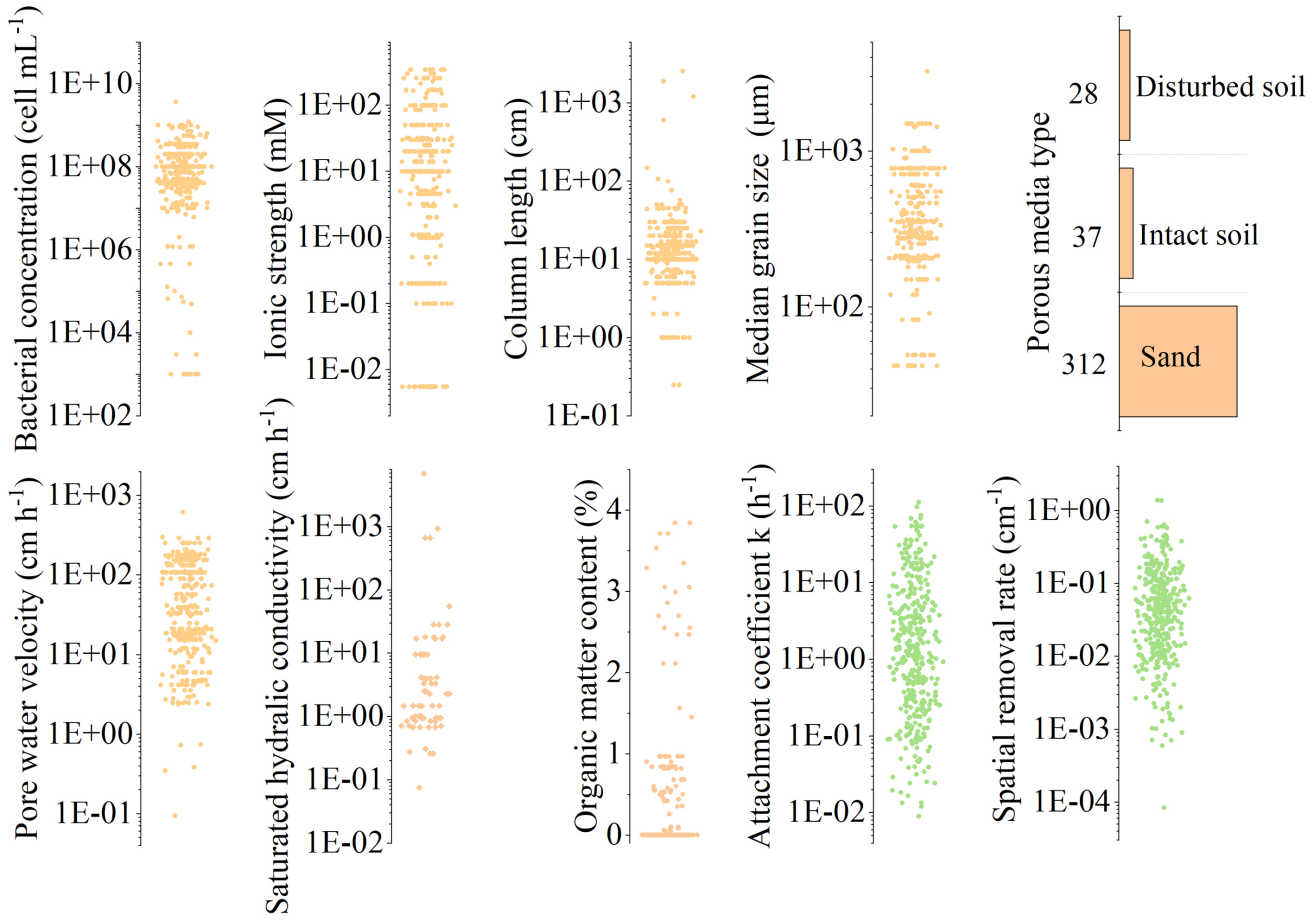
Statistics of model input and target variables datasets.

The column scatter plot of collected dataset is shown in Figure 2. For the input variables, the bacterial concentration ranged from 10^3^ to 10^9^ cell mL^−1^; the ionic strength of liquid phase was normalized as NaCl solution ranging from 10^−3^ to 10^3^ mM; the column length was ranged from 0.25 to 2,565 cm; the median grain sizes of porous media varied from 42 to 1,500 μm; the pore water velocity ranged from 0.09 to 618 cm h^−1^; the saturated hydraulic conductivity ranged from 0.08 to 55.8 cm h^−1^; the organic matter content ranged from 0.05 to 3.84%; the number of datasets for sand, intact soil, and disturbed soil was 314, 37, and 28, respectively. Because literature usually described particle size distribution of sand and soil in different ways, i.e., median grain size is usually used for sand; while soil texture is described by sand, silt, and clay percentage (here we use saturated hydraulic conductivity to reflect soil texture property). Thus, in our datasets, most sand lacked saturated hydraulic conductivity data and most soil lack median grain sizes data. Regarding the target variables, the first-order attachment coefficient (*k*) ranged from 0.009 to 111.6 h^−1^, and the spatial removal rate (λ) ranged from 0.00008 to 1.379 cm^−1^. Overall, the collected datasets covered the range in a variety of flow-through experiments.

**Figure 3:**
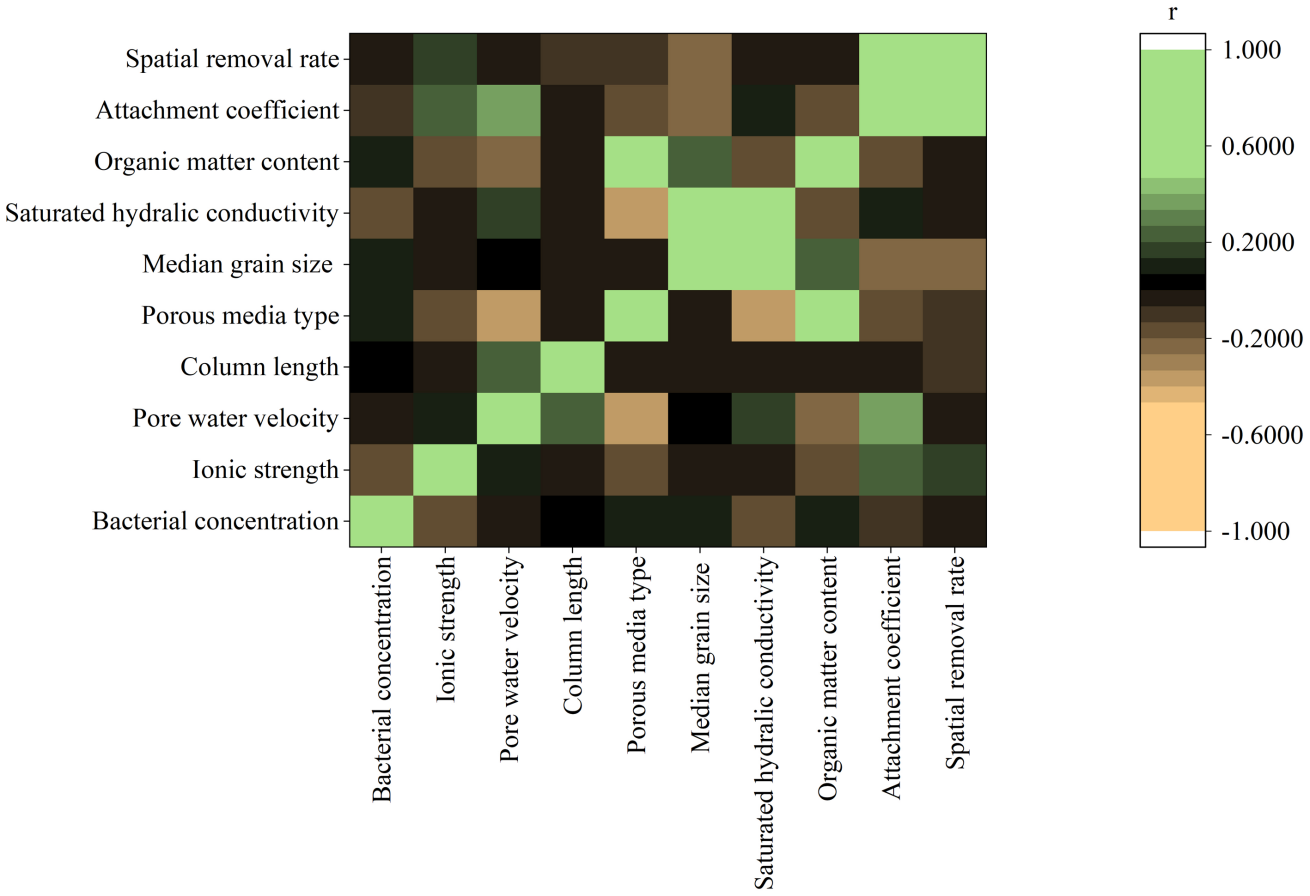
Pearson product-moment correlation coefficient (r) between pairwise variables (porous media type: 1-sand; 2-distubed soil; 3-intact soil).

The Pearson correlation coefficient (r) between pairwise variables is shown in Figure 3. The r values of *k* and *λ* with other eight input variables ranged from −0.25 to 0.34 and − 0.22 to 0.17, respectively. The weak Pearson correlations imply that the eight input variables could not be a valid predictor independently. The r between *k* and *λ* was 0.68, indicating that these two target variables were moderately correlated in positive manner (*k* = *λv*). However, when *v* varies, the increased *k* does not necessarily indicate an increase in *λ*.

Determination of variables and data collection are the two key prerequisites for training machine learning models. In the study, two types of factors were not considered. The first type of factors has small differences but may influence bacterial transport behaviors. For example, cell surface characteristics (e.g., physiological state, flagella type, and extracellular polymeric substances) were reported to affect bacterial transport in sand or soil (Madumathi et al., 2017; Du et al., 2021; Zhang et al., 2021). These variables were not included into the AML model because they were not clearly defined in most literature. The second type may affect bacterial transport, but the effects are minor, such as temperature and bacterial starvation (Kim and Walker, 2009; Walczak et al., 2012).

### 3.2. Model performance

**Figure 4:**
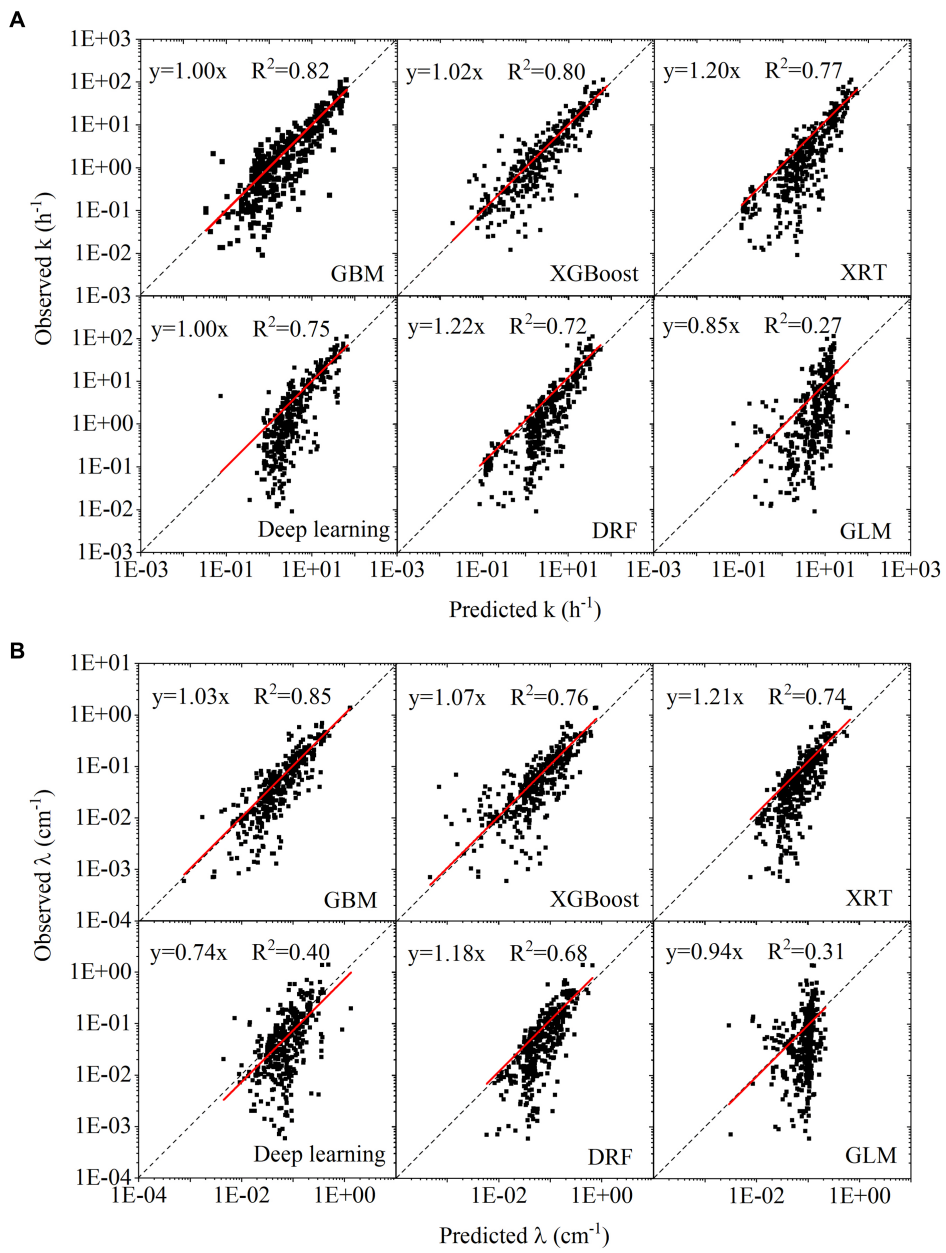
**(A)** Linear regression between observed target variables and predicted target variables for first-order attachment coefficient ( k ); **(B)** linear regression between observed target variables and predicted target variables for spatial removal rate ( λ ).

Based on the results of 5-fold cross-validation, the correlation between predicted values and observed values for the test datasets was plotted in Figures 4A,B. During the H2O AutoML training process, 20 models (six types of machine learning algorithms) were trained simultaneously, and their ranking based on RMSE and MAE was listed in Supplementary Table S2. For the predictions of *k* and *λ*, the best model was GBM, followed by XGboost. The slope of linear fitting in GBM was close to 1. The *R*^2^ of linear regression using GBM was 0.82 and 0.85 in Figures 4A,B, respectively. XRT, Deep learning and DRF showed similar performance, belonging to the second tier. GLM showed the worst performance among six machine learning algorithms.

The data points were plotted in log scale, some negative values in predicted values cannot be shown in figures. The summary of negative values was as follows. The number of negative values in predicting *k* with GBM, XGboost, XRT, Deep learning, DRF and GLM was 14, 31, 0, 9, 0, and 17, respectively. The number of negative values in predicting *λ* with GBM, XGboost, XRT, Deep learning, DRF and GLM was 4, 19, 0, 4, 0, and 13, respectively. The numbers of negative values indicated that although XGboost showed similar performance as GBM in the statistical measures (RSME, MAE, *R*^2^), many negative predicted values greatly affected its reliability. Compared to the prediction of *k*, prediction of *λ* showed fewer negative values.

Although GBM showed the best model performance, the hyperparameters affected the model performance greatly. In Supplementary Table S2, the number that attached to each machine learning algorithm refers to different hyperparameter settings, which can be extracted from the H2O AutoML for further tuning in a regular machine learning model. For example, the GBM_5 was ranked first while the GBM_1 was ranked eighteenth for predicting *k*. The RMSE of GBM_1 was two times of that of GBM_5. Compared with regular machine learning model training, the automatically set hyperparameters in the H2O AutoML significantly improved the efficiency of model training (LeDell and Poirier, 2020)x.

**Figure 5:**
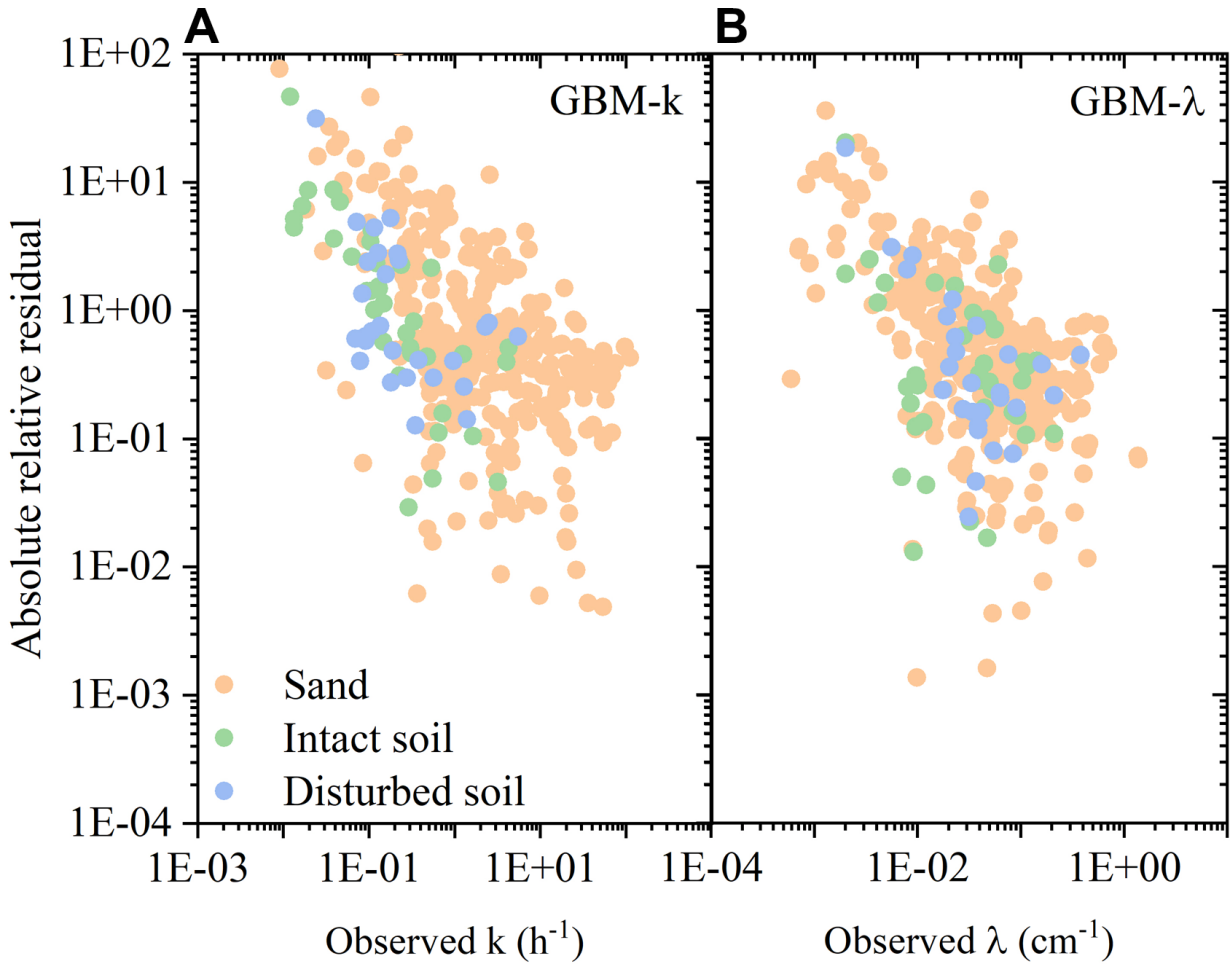
**(A)** Absolute relative residual in GBM predictive model for predicting first-order attachment coefficient ( k ); **(B)** Absolute relative residual in GBM predictive model for predicting spatial removal rate ( λ ).

To better analyze the results from predictive models, we chose the best performed model GBM to continue the statistical analysis. The absolute relative residuals (ARR) in GBM predictive model for predicting *k* and *λ* are shown in Figures 5A,B. Because the target variable values were widely distributed (cover 4–5 orders of magnitude), the basic statistical measures (RMSE, MAE, *R*^2^) cannot reflect the errors of all the values equally. In contrast, the ARR value can provide more information (Li et al., 2020). As the increase of observed values, the ARR decreased. Namely, when *k* and *λ* was high, the ARR was relatively small. Specifically, for scenarios with higher bacterial retention, such as smaller median grain size, higher ionic strength, and longer bacterial transport distance, the predictive models showed better performance. Compared to the ARR values for predicting *k*, that for predicting *λ* were slightly lower. It indicates that predicting *λ* is a better choice than predicting k. Besides, in Figures 5A,B, the ARR values of three types of porous media were separately plotted. It showed that there was no obvious difference among sand, intact soil, and disturbed soil.

### 3.3. Analysis of variable importance

**Figure 6:**
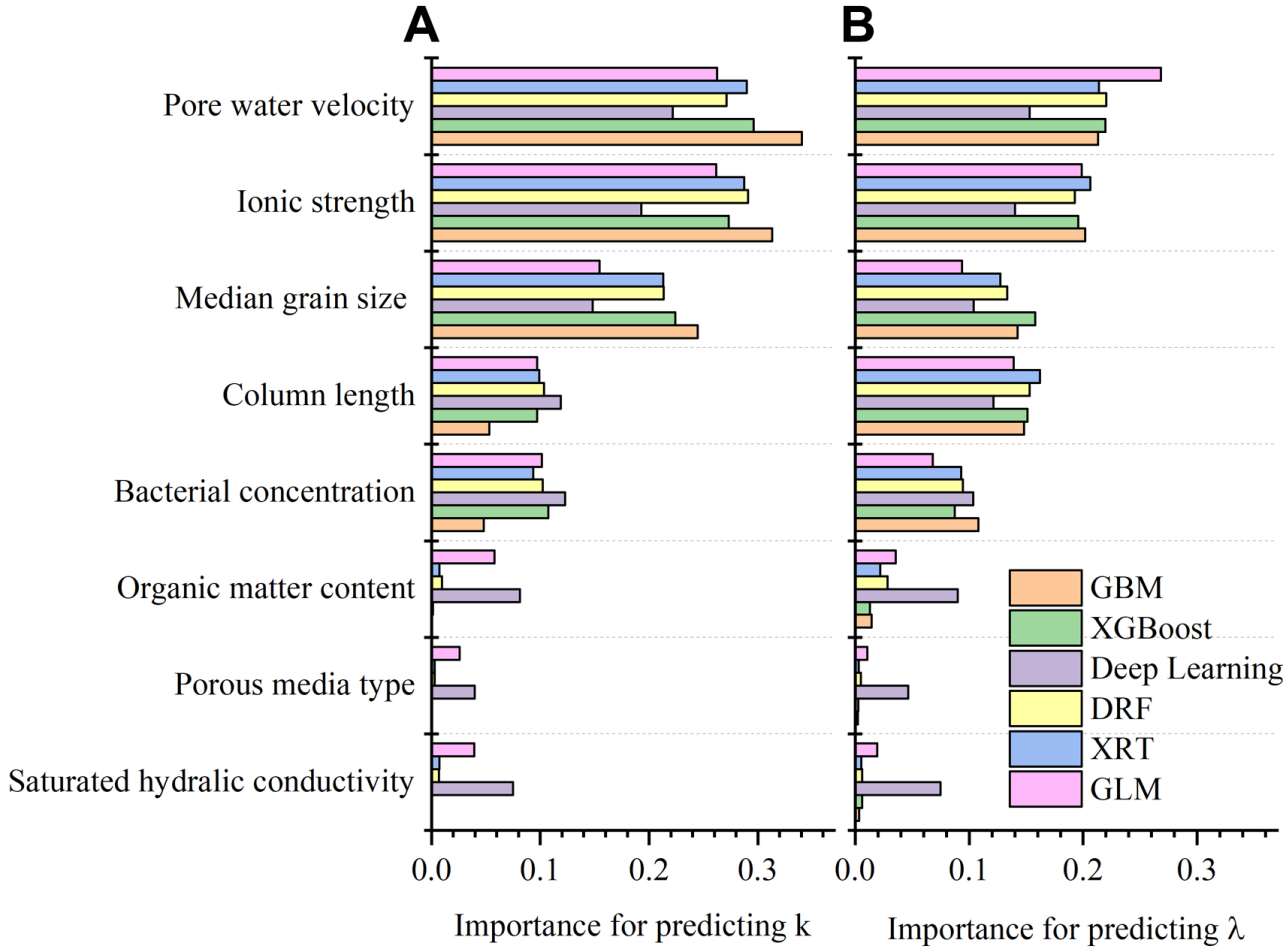
**(A)** Variable importance in six predictive models for predicting first-order attachment coefficient ( k ); **(B)** variable importance in six predictive models for predicting spatial removal rate ( λ ).

The importance of variables in six types of machine learning algorithms is shown in Figures 6A,B. The variable importance for predicting *k* and *λ* was similar, ranking as pore water velocity = ionic strength > median grain size > column length > bacterial concentration > organic content > porous medium type = saturated hydraulic conductivity.

**Figure 7:**
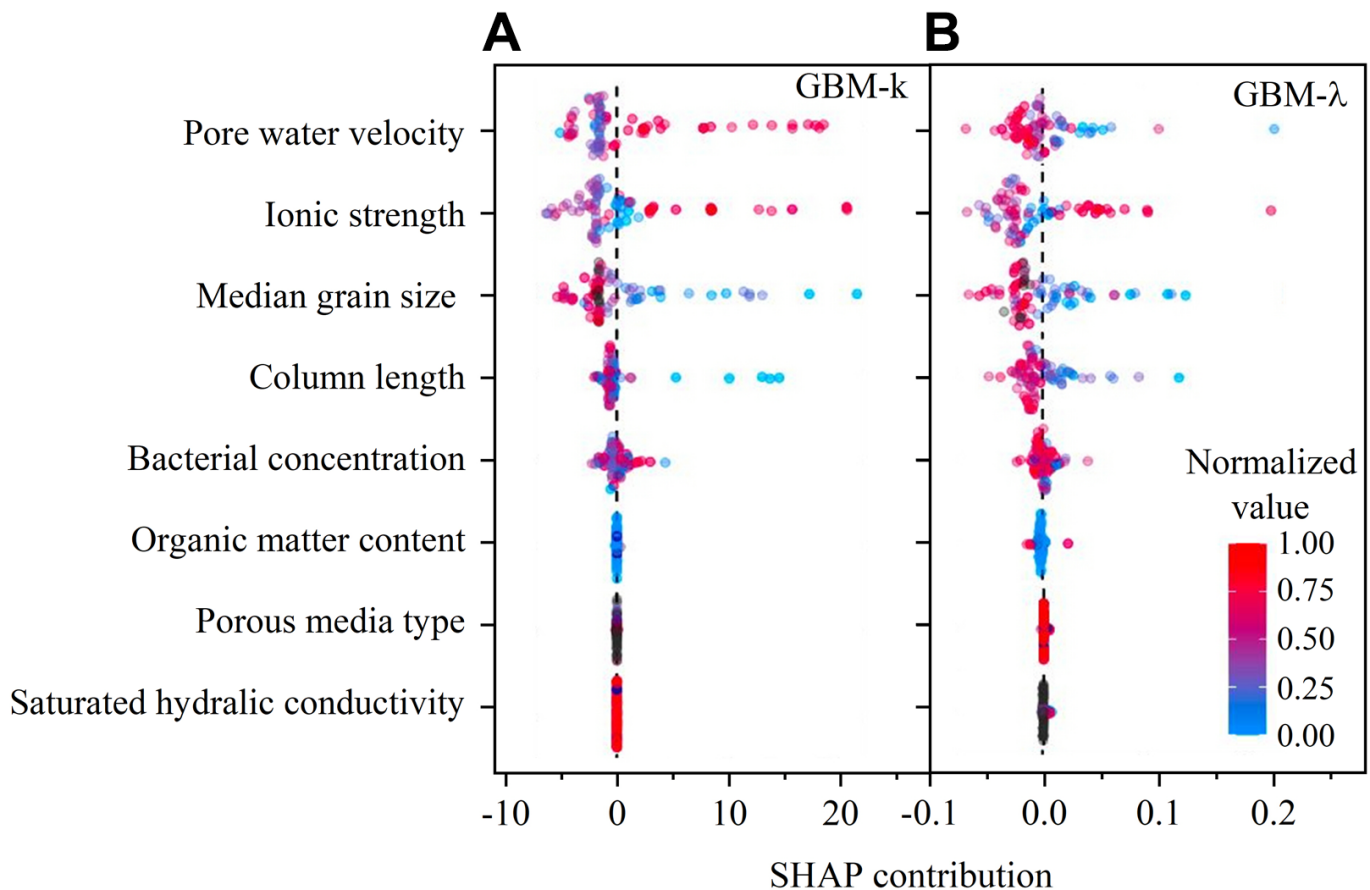
**(A)** SHAP contribution in GBM predictive model for predicting first-order attachment coefficient ( k ); **(B)** SHAP contribution in GBM predictive model for predicting spatial removal rate ( λ ).

The SHAP contribution of GBM is demonstrated in Figures 7A,B. In the SHAP summary plot, the color represents the normalized values of each data point in the testing dataset. The SHAP contribution value represents the positive or negative contribution of each data point for predicting target variables. As shown in the SHAP summary plot, larger median grain size and column length contributed to smaller *k* and λ value, larger ionic strength led to bigger *k* and λ value, and bacterial concentration, organic matter content, porous medium type and saturated hydraulic conductivity did not influence *k* and λ value. Besides, pore water velocity showed opposite effect on *k* and λ value because of the inverse relation (i.e., k=λv ) between *k* and λ when pore water velocity is a constant.

Pore water velocity showed highest importance in the predictions of *k* and *λ*. This is because bacterial transport could be increased by increasing pore water velocity (Bradford et al., 2006; Choi et al., 2007; Chen et al., 2022). Higher pore water velocity is accompanied by higher water shear force and less bacteria-soil contact time, which can reduce bacterial mechanical filtration and bacterial attachment, respectively (Hendry et al., 1999; Li et al., 2005; Syngouna and Chrysikopoulos, 2011). The responses of *k* and *λ* to pore water velocity agree with the filtration theory (Logan et al., 1995). Ionic strength is another important variable. Higher ionic strength favored bacterial retention as manifested by larger *k* and *λ* values. The responses of *k* and *λ* to solution ionic strength agree with the DLVO theory (Kim and Walker, 2009; Walczak et al., 2012; Wang et al., 2013; Bai et al., 2017).

Median grain size of porous media is correlated to soil texture and soil porosity. The bigger median grain size of porous media was favorable to reduce bacterial retention (i.e., smaller *k* and *λ*). This trend has been well confirmed in previous studies (Gannon et al., 1991; Balkhair, 2017; Sepehrnia et al., 2017). The effect of soil column length can be regarded as a scale effect (Hijnen et al., 2005; Knappett et al., 2014). As shown in the SHAP contribution figures, the upscaling of bacterial transport resulted in smaller *k* and *λ*. The distribution of column length data points was more scattered for predicting *λ* than predicting *k*, suggesting that *λ* is more sensitive to column length. This result is consistent with a recent study that the upscaling effect is more pronounced for *λ* than *k* (Oudega et al., 2021).

For porous medium type, bacterial concentration, organic content, and saturated hydraulic conductivity, the SHAP contribution shows relatively aggregated distribution. Many studies showed that the intact soil could greatly facility bacterial transport because of preferential flow in macropore-dominated pathways (McLeod et al., 1998; Safadoust et al., 2011; Chen et al., 2022). Nevertheless, the effect of intact soil or disturbed soil was not obvious in our predictive models. The reason may be the database of soil was small, which is not enough for distinguishing the contribution of intact or disturbed soil. Similarly, the contribution of organic content, and saturated hydraulic conductivity faced with the same problem (small database limited the variable importance analysis). Previous studies showed that increase in bacterial concentration may either increase or decrease bacterial retention by blocking or ripening, respectively (Bradford and Bettahar, 2006; Zhang et al., 2010). This concentration effect may also be related to solution ionic strength (Bradford et al., 2009). Therefore, it is not possible to conclude the positive or negative contribution of bacterial concentration to the transport.

### 3.4. Comparison of different machine learning algorithms

Among six machine learning algorithms, GBM and XGBoost belongs to gradient boosting algorithms; DRF and XRT are random forest-based algorithms; Deep Learning is based on artificial neural networks; GLM is a flexible generalization of ordinary linear regression (Mahesh, 2020). From the perspective of regression performance, the algorithms based on gradient boosting outperformed other algorithms, because they can optimize on different loss functions to make the function fit very flexible. Thus, they have higher accuracy than random forest-based algorithms, artificial neural networks and generalized linear model (Madeh Piryonesi and El-Diraby, 2021). The GLM always showed the worst performance, implying that GLM has weak ability to predict when the problem is complicated (many variables).

Although GBM and XGBoost showed better performance than other algorithms, some predicted values from them were negative, which violates the physics of bacterial transport and retention in porous media. In contrast, the random forest-based algorithms, such as DRF and XRT, are more consistent with physical laws. Therefore, to predict bacterial transport parameters, different machine learning algorithms may be used in a combination way. For example, under most conditions, GBM and XGBoost are suitable. Once the negative values appear, the random forest-based algorithms, such as DRF and XRT can be used as a supplement.

### 3.5. Limitations and future application

The main limitation of the predictive models is that the datasets of soil in database is small (17.2%), which makes that predictive models were not sensitive to porous medium type, saturated hydraulic conductivity, and organic matter content. This study only collected data from the transport of *E coli*. Therefore, the data in literature was not enough. Further studies, such as building a bigger database comprising of different bacteria or microorganisms, may provide more extensive and accurate predictive models.

Compared to previous studies based on controlled variables, the data-driven machine learning algorithms provides an advantageous approach for regression problems. With machine learning algorithms, many input variables that have low correlations with the target variables can predict the target variables with very high accuracy. This extraordinary performance of the AML model has been confirmed in other studies (Wilson et al., 2020; Qi et al., 2021; Fallatah et al., 2022; Xu et al., 2022).

The collected datasets and R code for the H2O AutoML are shown in the Supplementary material. The users may use the collected datasets and their own data to train an AML model and then use it to predict transport parameters for *E. coli* under saturated flow conditions. When combined with mechanism-based models and software, such as Hydrus, the bacterial transport can be simulated and visualized (Šimunek et al., 2012). The users may also add more variables to expand to a more comprehensive prediction for solute and colloid transport in the vadose zone.

## 4. Conclusion

In this study, literature-based data regarding *E. coli* transport through saturated sand or soil were used to train an AML model (H2O AutoML). We used bacterial concentration, porous medium type, median grain size, ionic strength, pore water velocity, column length, saturated hydraulic conductivity, and organic matter content as input variables to predict first-order attachment coefficient (*k*) and spatial removal rate (*λ*). The results showed that the trained machine learning models were reliable tools to predict key parameters for *E. coli* transport through saturated porous media. Among six types of machine learning algorithms, the gradient boosted based algorithms, such as Gradient Boosting Machine and Extreme Gradient Boosting, outperformed other machine learning algorithms. The predictive models showed better performance when bacterial retention was high. Besides, spatial removal rate is a better target variable than first-order attachment coefficient. Compared with traditional controlled variable experiments, the data-driven AML accomplished the goal that predicting bacterial transport from a comprehensive perspective. This approach offers a new way of thinking for predicting environmental fates of various pollutants.

## Data availability statement

The original contributions presented in the study are included in the article/Supplementary material, further inquiries can be directed to the corresponding author.

## Author contributions

JZ and XC contributed to conception and design of the study. FC organized the database, performed the statistical analysis, and wrote the first draft of the manuscript. BZ, LY, JZ, and XC reviewed the manuscript. All authors contributed to the article and approved the submitted version.

## Funding

This work was financially supported by the National Natural Science Foundation of China (Grant No. 41730858), the Strategic Priority Research Program of the Chinese Academy of Sciences (Grant No. XDA28090100), and the Liaoning Science and Technology Plan Project (Grant No. 2021JH2/10300079).

## Conflict of interest

The authors declare that the research was conducted in the absence of any commercial or financial relationships that could be construed as a potential conflict of interest.

## Publisher’s note

All claims expressed in this article are solely those of the authors and do not necessarily represent those of their affiliated organizations, or those of the publisher, the editors and the reviewers. Any product that may be evaluated in this article, or claim that may be made by its manufacturer, is not guaranteed or endorsed by the publisher.

## Supplementary material

The Supplementary material for this article can be found online at: https://www.frontiersin.org/articles/10.3389/fmicb.2023.1152059/full#supplementary-material
