# Supplementary material for: Predicting bacterial transport through saturated porous media using an automated machine learning model
Source: Front Microbiol. 2023 May 10;14:1152059. doi: 10.3389/fmicb.2023.1152059 (PMC10206036; doi:10.3389/fmicb.2023.1152059)
Supplement: Supplementary file 1 [file Data_Sheet_1.pdf]

Supplementary Material

Table S1: Collected datasets

Paper No.	Concentration (cell/ml)	Ionic strength (mM)	Velocity (cm/h)	L (cm)	Porous media Type	Median grain size (µm)	Saturated hydralic conductivity (cm/h)	Organic matter content (%)	k temporal removal rate (h-1)	λ spatial removal rate (cm-1)
1	1.25E+07	1	16.67	10	sand	335		0	0.17563598	0.01053605
	1.25E+07	10	16.67	10	sand	335		0	0.25142176	0.01508229
	1.25E+07	100	16.67	10	sand	335		0	0.3719803	0.02231436
2	1.60E+07	100	31.84	10	sand	363		0	2.08210187	0.06539265
3	1.00E+08	100	8.7	10	sand	440		0	1.20607609	0.13862944
	1.00E+08	100	13	10	sand	440		0	0.90109133	0.06931472
4	2.00E+08	0.0055	15.3	30	sand	1000		0	0.18	0.01176471
	2.00E+08	0.0055	15.3	30	sand	1000		0	0.216	0.01411765
	2.00E+08	0.0055	15.3	30	sand	1000		0	0.138	0.00901961
	2.00E+08	0.0055	15.3	30	sand	1000		0	0.102	0.00666667
5	1.00E+08	10	4.16	11	sand	213		0	0.6894	0.16572115
	1.00E+08	10	20.8	11	sand	213		0	1.44	0.06923077
	1.00E+08	10	208	11	sand	213		0	1.4832	0.00713077
	1.00E+08	30	4.16	11	sand	213		0	1.368	0.32884615
	1.00E+08	30	20.8	11	sand	213		0	1.602	0.07701923
	1.00E+08	30	208	11	sand	213		0	2.94	0.01413462

	1.00E+08	50	4.16	11	sand	213		0	1.74	0.41826923
	1.00E+08	50	20.8	11	sand	213		0	2.94	0.14134615
	1.00E+08	50	208	11	sand	213		0	5.04	0.02423077
6	1.20E+07	10	16.67	10	sand	509		0	0.33081771	0.01984509
	1.20E+07	10	16.67	10	sand	509		0	1.22352661	0.07339692
7	1.00E+03	10	8.03	25	Disturbed soil	320	28.5	0.68	0.07162908	0.00892574
	1.00E+03	10	16.05	25	Disturbed soil	320	28.5	0.68	0.1274055	0.00793804
	1.00E+03	10	32.10	25	Disturbed soil	320	28.5	0.68	0.17881249	0.00557048
	1.00E+03	10	8.03	25	sand	400		0	0.09234595	0.01150728
	1.00E+03	10	16.05	25	sand	400		0	0.22898531	0.014267
	1.00E+03	10	32.10	25	sand	400		0	0.6559001	0.02043302
8	1.25E+07	10	33.3	10	sand	363		0	0.35085052	0.01053605
	1.25E+07	50	33.3	10	sand	363		0	2.30818011	0.06931472
9	1.00E+08	1	74.64	20	sand	600		0	0.31118016	0.00416908
	1.00E+08	20	74.64	20	sand	600		0	0.39320544	0.00526803
	1.00E+08	100	74.64	20	sand	600		0	0.56287102	0.00754114
10	1.00E+08	0.1	36.3	17	sand	1050		0	2.07734455	0.05722712
11	1.00E+08	150	4.77	15	sand	363		0	0.16297299	0.03416625
	1.00E+08	150	4.77	15	sand	256		0	0.19011217	0.0398558
	1.00E+08	150	4.77	15	sand	128		0	0.22361681	0.04687983
	1.00E+08	150	4.77	15	sand	91		0	0.25605255	0.05367978

12	2.00E+08	10	33.3	10	sand	400	0	0.97578883	0.02930297
	2.00E+08	50	33.3	10	sand	400	0	1.57578517	0.04732088
13	1.35E+07	5	16.67	10	sand	550	0	0.47956601	0.02876821
	1.35E+07	25	16.67	10	sand	550	0	0.71811512	0.04307829
	1.35E+07	5	16.67	10	sand	550	0	0.08550592	0.00512933
	1.35E+07	25	16.67	10	sand	550	0	0.25142176	0.01508229
	1.35E+08	5	16.67	10	sand	550	0	0.3719803	0.02231436
	1.35E+08	25	16.67	10	sand	550	0	0.54761628	0.03285041
	1.35E+08	5	16.67	10	sand	550	0	1.09009542	0.06539265
	1.35E+08	25	16.67	10	sand	550	0	1.33111233	0.07985077
14	4.07E+07	17.11	108	12	sand	150	0	1.18123458	0.01093736
	4.23E+07	85.55	108	12	sand	150	0	3.17158548	0.02936653
	3.97E+07	171.1	108	12	sand	150	0	19.9476666	0.18470062
	3.81E+07	256.65	108	12	sand	150	0	31.864135	0.29503829
	4.33E+07	85.55	108	12	sand	150	0	4.77466431	0.04420985
	4.11E+07	171.1	108	12	sand	150	0	13.0335279	0.12068081
	3.91E+07	256.65	108	12	sand	150	0	16.1619074	0.14964729
	3.03E+07	256.65	108	12	sand	150	0	16.7210934	0.15482494
	4.42E+07	17.11	108	12	sand	350	0	0.09045302	0.00083753
	3.72E+07	42.775	108	12	sand	350	0	0.24634077	0.00228093
	4.14E+07	85.55	108	12	sand	350	0	0.66281886	0.00613721

4.37E+07	128.325	108	12	sand	350	0	0.47111832	0.00436221
3.81E+07	171.1	108	12	sand	350	0	1.13006901	0.0104636
2.54E+07	213.875	108	12	sand	350	0	1.11987071	0.01036917
4.62E+07	230.985	108	12	sand	350	0	3.01925463	0.02795606
3.42E+07	256.65	108	12	sand	350	0	4.3459763	0.04024052
3.62E+07	256.65	108	12	sand	350	0	4.3751971	0.04051108
4.72E+07	342.2	108	12	sand	350	0	8.405511	0.07782881
3.71E+07	85.55	108	12	sand	350	0	0.9682669	0.00896543
4.81E+07	171.1	108	12	sand	350	0	1.66612936	0.01542712
2.97E+07	256.65	108	12	sand	350	0	2.21310485	0.02049171
3.57E+07	342.2	108	12	sand	350	0	3.57747245	0.03312474
3.13E+07	342.2	108	12	sand	350	0	7.14665789	0.06617276
4.01E+07	342.2	108	12	sand	350	0	5.76499257	0.05337956
4.37E+07	17.11	108	12	sand	710	0	0.0090045	8.3375E-05
3.58E+07	171.1	108	12	sand	710	0	1.87429445	0.01735458
4.01E+07	256.65	108	12	sand	710	0	4.4634331	0.04132808
3.61E+07	342.2	108	12	sand	710	0	3.94654466	0.03654208
3.65E+07	85.55	108	12	sand	710	0	1.50512327	0.01393633
2.38E+07	171.1	108	12	sand	710	0	2.1101158	0.01953811
2.93E+07	256.65	108	12	sand	710	0	2.121501	0.01964353
4.33E+07	342.2	108	12	sand	710	0	4.3751971	0.04051108

	2.17E+07	342.2	108	12	sand	710	0	5.18628086	0.04802112
15	1.50E+07	20	16.67	10	sand	319	0	0.50194219	0.03011051
16	3.00E+08	1	2.93	12	sand	205	0	0.05448422	0.0185953
	3.00E+08	10	2.93	12	sand	205	0	0.07024237	0.02397351
	3.00E+08	100	2.93	12	sand	205	0	0.22372765	0.07635756
17	9.08E+08	0.1	71.31	17	sand	395	0	3.20314258	0.04491586
	5.62E+08	0.1	73.99	17	sand	395	0	3.45628113	0.04671018
	7.08E+08	0.1	57.58	17	sand	1030	0	3.1721728	0.05508785
	8.40E+08	0.1	57.72	17	sand	1030	0	2.96179119	0.05131023
18	5.00E+07	25	16.67	10	sand	509	0	1.09009542	0.06539265
19	1.00E+07	10	33.3	20	sand	509	0	0.47899065	0.0143841
	1.00E+07	20	33.3	20	sand	509	0	0.74306803	0.02231436
	1.00E+07	30	33.3	20	sand	509	0	1.05707232	0.03174391
20	2.50E+07	3	133	7.35	sand	275	0	1.44	0.01082707
	2.50E+07	10	133	7.35	sand	275	0	2.16	0.0162406
	2.50E+07	30	133	7.35	sand	275	0	10.8	0.08120301
	2.50E+07	50	133	7.35	sand	275	0	18	0.13533835
	2.50E+07	100	133	7.35	sand	275	0	46.8	0.3518797
	2.50E+07	300	133	7.35	sand	275	0	54	0.40601504
	2.50E+07	10	133	7.35	sand	275	0	1.512	0.01136842
	2.50E+07	30	133	7.35	sand	275	0	2.52	0.01894737

	2.50E+07	50	133	7.35	sand	275	0	7.2	0.05413534
	2.50E+07	100	133	7.35	sand	275	0	36	0.27067669
	2.50E+07	300	133	7.35	sand	275	0	54	0.40601504
21	1.70E+08	14	5.57	15	sand	710	0	0.09129242	0.01639337
	1.70E+08	14	19.16	15	sand	710	0	0.24726791	0.01290565
	1.70E+08	14	35.64	15	sand	710	0	0.52716714	0.01479296
	1.70E+08	14	4.22	15	sand	360	0	0.17191206	0.04070973
	1.70E+08	14	17.84	15	sand	360	0	0.37425062	0.02098072
	1.70E+08	14	33.79	15	sand	360	0	0.86553819	0.02561286
	1.70E+08	14	4.81	15	sand	240	0	0.76586585	0.15906445
	1.70E+08	14	17.53	15	sand	240	0	1.01104671	0.05767483
	1.70E+08	14	3.05	10	sand	150	0	1.77404966	0.5809143
	1.70E+08	14	17.95	15	sand	150	0	2.42281809	0.13499689
	1.70E+08	14	30.34	10	sand	150	0	2.29095424	0.07550226
22	1.00E+08	20	41.67	6.8	sand	280	0	0.49	0.01176
	1.00E+08	20	41.67	6.8	sand	280	0	0.54	0.01296
	1.00E+08	20	41.67	6.8	sand	280	0	0.7	0.0168
	1.00E+08	20	20.83	6.8	sand	280	0	0.29	0.01392
	1.00E+08	20	20.83	6.8	sand	280	0	0.27	0.01296
	1.00E+08	20	20.83	6.8	sand	280	0	0.29	0.01392
	1.00E+08	20	4.17	6.8	sand	280	0	0.1	0.024

	1.00E+08	20	4.17	6.8	sand	280	0	0.05	0.012
	1.00E+08	20	4.17	6.8	sand	280	0	0.04	0.0096
23	1.00E+08	5	4.17	5	sand	213	0	0.08965434	0.02151704
	1.00E+08	10	4.17	5	sand	213	0	0.25770521	0.06184925
	1.00E+08	20	4.17	5	sand	213	0	0.33124745	0.07949939
24	1.15E+06	20	12	10	sand	420	0	0.37765289	0.03147107
25	4.00E+07	165	18.6	15	sand	774	0	3.438	0.18483871
	4.00E+07	165	18.6	15	sand	774	0	3.06	0.16451613
26	1.94E+08	2	78.46	30	sand	513	0	0.1259056	0.00160468
	1.98E+08	2	24.62	30	sand	1440	0	0.03435012	0.00139547
	2.00E+08	2	24.62	30	sand	513	0	0.0464167	0.00188568
	1.88E+08	2	24.62	30	sand	181	0	0.03178735	0.00129136
27	4.00E+07	8.4	18.6	15	sand	780	0	0.05061927	0.00272147
	4.00E+07	168	18.6	15	sand	780	0	0.53417082	0.02871886
	4.00E+07	8.4	18.6	15	sand	780	0	0.02505136	0.00134685
	4.00E+07	168	18.6	15	sand	780	0	0.57292397	0.03080236
28	4.00E+07	1	18.6	15	sand	774	0	0.51523915	0.02770103
	4.00E+07	3	18.6	15	sand	774	0	0.8595025	0.04620981
	4.00E+07	10	18.6	15	sand	774	0	1.01801588	0.05473204
	4.00E+07	30	18.6	15	sand	774	0	1.0757007	0.05783337
	4.00E+07	100	18.6	15	sand	774	0	0.91012178	0.04893128

	4.00E+07	1	18.6	15	sand	774	0	0.55339601	0.02975247
	4.00E+07	3	18.6	15	sand	774	0	0.91012178	0.04893128
	4.00E+07	10	18.6	15	sand	774	0	0.936228	0.05033484
	4.00E+07	30	18.6	15	sand	774	0	1.0757007	0.05783337
	4.00E+07	100	18.6	15	sand	774	0	1.04652289	0.05626467
	4.00E+07	1	18.6	15	sand	774	0	0.276698	0.01487624
	4.00E+07	3	18.6	15	sand	774	0	0.55339601	0.02975247
	4.00E+07	10	18.6	15	sand	774	0	0.51523915	0.02770103
	4.00E+07	30	18.6	15	sand	774	0	0.59276439	0.03186905
	4.00E+07	100	18.6	15	sand	774	0	0.59276439	0.03186905
	4.00E+07	1	18.6	15	sand	774	0	0.276698	0.01487624
	4.00E+07	3	18.6	15	sand	774	0	0.63342377	0.03405504
	4.00E+07	10	18.6	15	sand	774	0	0.49659218	0.0266985
	4.00E+07	30	18.6	15	sand	774	0	0.61292744	0.03295309
	4.00E+07	100	18.6	15	sand	774	0	0.55339601	0.02975247
29	2.00E+08	5.3	10.6	30	sand	1000	0	0.366	0.0345283
30	5.00E+07	10	39.6	10	sand	205	0	2.46	0.06212121
	5.00E+07	10	39.6	10	sand	205	0	3.6	0.09090909
	5.00E+07	30	39.6	10	sand	205	0	7.26	0.18333333
	5.00E+07	30	39.6	10	sand	205	0	5.4	0.13636364
	5.00E+07	50	39.6	10	sand	205	0	17.4	0.43939394

	5.00E+07	50	39.6	10	sand	205	0	15	0.37878788
	5.00E+07	100	39.6	10	sand	205	0	54	1.36363636
	5.00E+07	100	39.6	10	sand	205	0	54.6	1.37878788
31	4.15E+08	0.0055	300.76	44	sand	1430	0	0.79655609	0.0026485
	9.25E+08	0.0055	99.03	22	sand	1430	0	1.00441722	0.01014289
	5.55E+08	0.0055	20.22	44	sand	288	0	4.97297674	0.24590405
	3.74E+08	0.0055	25.03	44	sand	288	0	4.61460166	0.18435746
	4.80E+08	0.0055	58.29	22	sand	288	0	4.63292958	0.07948636
	4.21E+08	0.0055	47.24	22	sand	288	0	9.13570404	0.19338697
32	1.00E+08	10	33.3	10	sand	300	0	7.66760836	0.23025851
	1.00E+08	1	33.3	10	sand	300	0	6.54715581	0.19661129
33	9.30E+07	1	175.8	13.6	sand	205	0	0.396	0.00225256
	8.30E+07	1	175.8	14.2	sand	205	0	0.1044	0.00059386
	1.10E+08	1	175.8	14	sand	205	0	0.612	0.00348123
	3.20E+07	1	175.8	22.5	sand	205	0	0.2916	0.0016587
	6.00E+07	3.2	175.8	3.2	sand	205	0	0.864	0.00491468
	9.10E+07	3.2	175.8	11.9	sand	205	0	0.54	0.00307167
	3.70E+07	3.2	175.8	11.9	sand	205	0	0.504	0.00286689
	5.80E+07	3.2	175.8	11.7	sand	205	0	0.648	0.00368601
	2.50E+07	10	175.8	10	sand	205	0	2.412	0.01372014
	1.00E+08	10	175.8	14.1	sand	205	0	1.836	0.01044369

	7.90E+07	10	175.8	14.2	sand	205		0	1.332	0.00757679
	8.30E+06	10	175.8	9.2	sand	205		0	1.476	0.0083959
	1.10E+07	10	175.8	9.4	sand	205		0	2.268	0.01290102
	1.00E+08	31.6	175.8	31.6	sand	205		0	8.28	0.04709898
	6.20E+06	31.6	175.8	14.5	sand	205		0	10.8	0.06143345
	1.00E+08	31.6	175.8	13.8	sand	205		0	5.04	0.02866894
	2.00E+06	31.6	175.8	13.6	sand	205		0	9.72	0.0552901
	9.10E+06	31.6	175.8	8.9	sand	205		0	8.28	0.04709898
	8.20E+06	31.6	175.8	9.1	sand	205		0	10.08	0.05733788
	1.30E+07	31.6	175.8	9.2	sand	205		0	6.48	0.03686007
	8.40E+07	100	175.8	100	sand	205		0	68.4	0.3890785
	9.80E+07	100	175.8	13.5	sand	205		0	75.6	0.43003413
	1.10E+08	100	175.8	13.7	sand	205		0	57.6	0.32764505
	1.80E+07	100	175.8	7.3	sand	205		0	68.4	0.3890785
	7.10E+06	100	175.8	7.3	sand	205		0	54	0.30716724
	1.80E+07	300	175.8	13.5	sand	205		0	111.6	0.63481229
34	2.00E+08	2	3.55	6	sand	463		0	1.57339552	0.44321001
	2.00E+08	20	3.55	6	sand	463		0	2.07471342	0.58442632
	2.00E+08	100	3.55	6	sand	463		0	2.4848255	0.69995085
	2.00E+08	2	10.56	6	sand	463		0	1.21993904	0.11552453
	2.00E+08	20	10.56	6	sand	463		0	1.84768694	0.17497035

	2.00E+08	100	10.56	6	sand	463	0	2.66486481	0.25235462
	2.00E+08	2	21.3	6	sand	463	0	1.02127136	0.04794701
	2.00E+08	20	21.3	6	sand	463	0	2.05835566	0.09663642
	2.00E+08	100	21.3	6	sand	463	0	1.31727607	0.06184395
35	5.00E+07	30	90	10	sand	205	0	2.00829196	0.02231436
	5.00E+07	50	90	10	sand	205	0	7.59573063	0.08439701
	5.00E+07	100	90	10	sand	205	0	35.208207	0.3912023
	5.00E+07	30	90	10	sand	205	0	5.38053301	0.0597837
	5.00E+07	50	90	10	sand	205	0	12.4766493	0.13862944
	5.00E+07	100	90	10	sand	205	0	41.4465317	0.46051702
36	3.86E+07	0.2	195.48	5	sand	196	0	3.25988738	0.01667632
	4.67E+07	0.2	167.04	2	sand	196	0	8.79971027	0.05268026
	4.61E+07	0.2	194.76	1	sand	196	0	24.8968274	0.12783337
	1.78E+08	0.2	194.76	1	sand	196	0	20.520014	0.10536052
	1.20E+09	0.2	194.76	1	sand	196	0	9.98988201	0.05129329
	4.74E+07	0.2	190.44	1	sand	196	0	17.9605258	0.09431068
	4.32E+07	0.2	187.2	5	sand	83	0	6.52779081	0.03487068
	4.54E+07	0.2	184.32	2	sand	83	0	11.7811235	0.06391669
	6.45E+07	0.2	163.44	1	sand	83	0	6.67194678	0.04082199
	1.39E+08	0.2	184.32	1	sand	83	0	13.3762301	0.07257069
	5.90E+08	0.2	182.16	1	sand	83	0	5.54844924	0.03045921

4.69E+07	0.2	179.28	1	sand	83	0	5.46072672	0.03045921
4.22E+07	0.2	174.24	5	sand	49	0	11.9351023	0.06849806
4.63E+07	0.2	174.24	2	sand	49	0	21.6459536	0.12423068
4.34E+07	0.2	154.44	1	sand	49	0	36.4049572	0.23572233
4.83E+07	0.2	117.36	5	sand	49	0	9.40000944	0.08009551
4.46E+07	0.2	77.04	5	sand	49	0	6.63750317	0.08615658
1.00E+05	0.2	149.4	5	sand	49	0	12.4156015	0.08310309
1.00E+04	0.2	154.44	5	sand	49	0	17.9094337	0.1159637
1.00E+03	0.2	149.4	5	sand	49	0	15.2634696	0.10216512
1.22E+08	0.2	181.8	1	sand	49	0	27.4196014	0.15082289
3.67E+09	0.2	164.16	1	sand	49	0	19.1301913	0.11653382
1.77E+07	0.2	164.16	0.25	sand	49	0	54.7516997	0.33352644
4.27E+07	0.2	165.96	1	sand	49	0	28.9356881	0.17435339
4.41E+07	0.2	166.32	5	sand	42	0	27.3090971	0.16419611
3.76E+07	0.2	171	2	sand	42	0	24.5968172	0.14384104
3.77E+07	0.2	156.6	1	sand	42	0	49.2837026	0.31471074
4.95E+07	0.2	112.32	5	sand	42	0	12.2367513	0.10894544
4.89E+07	0.2	68.4	5	sand	42	0	10.6229138	0.15530576
1.30E+05	0.2	132.12	5	sand	42	0	30.9473391	0.2342366
1.00E+03	0.2	132.12	5	sand	42	0	24.2120663	0.18325815
1.56E+08	0.2	171	1	sand	42	0	36.0332964	0.21072103

	9.01E+08	0.2	172.08	1	sand	42	0	32.0635938	0.18632958
	4.80E+07	0.2	161.28	0.25	sand	42	0	97.2988626	0.60329156
	4.38E+07	0.2	166.32	1	sand	42	0	61.7153115	0.37106368
37	1.00E+08	5	18.6	20	sand	120	0	0.76351191	0.04104903
	1.00E+08	20	20.4	20	sand	120	0	2.16266881	0.10601318
	1.00E+08	100	22.2	20	sand	120	0	4.34234554	0.19560115
	1.00E+08	20	618	20	sand	710	0	0.62426366	0.00101014
	1.00E+08	100	618	20	sand	710	0	2.57649172	0.00416908
38	1.00E+08	3	15	23	sand	260	0	1.1183468	0.07455645
	1.00E+08	3	15	23	sand	260	0	0.93072806	0.06204854
39	6.91E+07	0.0055	156	10	sand	300	0	30.6713606	0.19661129
	7.18E+07	0.0055	156	15	sand	300	0	26.2675779	0.16838191
	6.71E+07	0.0055	156	20	sand	300	0	20.7422283	0.132963
	6.87E+07	0.0055	156	25	sand	300	0	17.5556829	0.11253643
	7.44E+07	0.0055	156	30	sand	300	0	15.5778078	0.09985774
	7.19E+07	0.0055	156	10	sand	600	0	24.3461049	0.15606477
	7.26E+07	0.0055	156	15	sand	600	0	21.2182966	0.13601472
	7.29E+07	0.0055	156	20	sand	600	0	17.2167443	0.11036375
	7.30E+07	0.0055	156	25	sand	600	0	15.0255806	0.09631782
	6.75E+07	0.0055	156	30	sand	600	0	13.133789	0.08419095
	7.28E+07	0.0055	156	10	sand	1000	0	21.626192	0.13862944

	7.27E+07	0.0055	156	15	sand	1000	0	19.7300478	0.12647467
	6.95E+07	0.0055	156	20	sand	1000	0	17.9601637	0.11512925
	7.15E+07	0.0055	156	25	sand	1000	0	15.7605467	0.10102915
	7.52E+07	0.0055	156	30	sand	1000	0	14.6297357	0.09378036
40	1.00E+09	10	110	37	sand	356	0	1.12796082	0.01025419
	1.00E+09	10	110	57	sand	356	0	0.86256103	0.00784146
	1.00E+09	10	110	77	sand	356	0	1.04979395	0.00954358
	1.00E+09	10	110	107	sand	356	0	0.90205233	0.00820048
	1.00E+09	10	110	147	sand	356	0	0.78962977	0.00717845
41	1.00E+08	10	292	600	sand	356	0	0.30202599	0.00103434
	1.00E+08	10	292	1215	sand	356	0	0.26272891	0.00089976
	1.00E+08	10	292	1900	sand	356	0	0.20791075	0.00071202
	1.00E+08	10	292	2565	sand	356	0	0.20493594	0.00070184
42	1.31E+08	10	2.76	25.4	sand	532	0	0.02910816	0.01054643
	1.57E+08	10	2.81	25.4	sand	532	0	0.01837061	0.00653758
43	3.00E+07	10	54	15	sand	387	0	3.69186159	0.06836781
	3.00E+07	100	54	15	sand	387	0	2.63110972	0.04872425
44	4.00E+07	1	50.4	15	sand	254	0	0.69499157	0.01378952
	4.00E+07	5	50.4	15	sand	254	0	1.0149002	0.02013691
	4.00E+07	20	50.4	15	sand	254	0	2.93322812	0.05819897
	4.00E+07	50	50.4	15	sand	254	0	7.91609208	0.15706532

45	9.08E+08	0.1	71.3142857	17	sand	360	0	3.58434618	0.05026126
	5.62E+08	0.1	73.9941691	17	sand	360	0	4.7850084	0.06466737
	6.29E+08	0.1	74.3023256	17	sand	360	0	3.73452885	0.05026126
	7.08E+08	0.1	57.5838926	17	sand	900	0	3.47939834	0.06042312
	8.40E+08	0.1	57.7232143	17	sand	900	0	3.39688208	0.05884776
46	1.00E+07	10	139.8	10	sand	607	0	0.57390755	0.0041052
	1.00E+07	10	139.8	10	sand	310	0	1.12052842	0.00801522
	1.00E+07	10	21	10	sand	607	0	0.20837461	0.0099226
	1.00E+07	10	21	10	sand	310	0	0.37978606	0.01808505
	1.00E+07	32	139.8	10	sand	607	0	1.39936018	0.01000973
	1.00E+07	32	139.8	10	sand	310	0	4.00465552	0.0286456
	1.00E+07	32	21	10	sand	607	0	0.50283	0.02394429
	1.00E+07	32	21	10	sand	310	0	1.69407045	0.08067002
47	1.00E+08	10	2.38	10	sand	300	0	0.31845995	0.1338067
	1.00E+08	10	2.38	10	sand	300	0	0.37110545	0.15592666
48	1.30E+08	1.5	108.3	15	sand	327	0	1.43281578	0.01323006
49	3.00E+08	1	252	30	sand	1500	0.82	1.74011385	0.00690521
	3.00E+08	5	252	30	sand	1500	0.82	3.61039909	0.01432698
	3.00E+08	10	252	30	sand	1500	0.82	4.46010069	0.01769881
	3.00E+08	50	252	30	sand	1500	0.82	6.09601761	0.02419055
	3.00E+08	100	252	30	sand	1500	0.82	7.35262584	0.02917709

	3.00E+08	1	252	30	sand	1500		0.9	2.47335535	0.0098149
	3.00E+08	50	252	30	sand	1500		0.9	9.05986673	0.03595185
	3.00E+08	1	252	30	sand	1500		0.05	2.48277146	0.00985227
	3.00E+08	50	252	30	sand	1500		0.05	3.66931527	0.01456077
50	2.00E+08	10	6	5	Intact soil		0.674	3.71	1.25978655	0.20996442
	2.00E+08	10	6	5	Intact soil		0.713	3.05	0.65367261	0.10894544
	2.00E+08	10	6	5	Intact soil		1.017	2.55	0.23814113	0.03969019
	2.00E+08	10	6	5	Intact soil		0.857	2.11	0.0120604	0.00201007
	2.00E+08	10	6	5	Disturbed soil		0.674	3.84	2.27654398	0.379424
	2.00E+08	10	6	5	Disturbed soil		0.713	3.35	0.95820924	0.15970154
	2.00E+08	10	6	5	Disturbed soil		1.017	2.86	0.37765289	0.06294215
	2.00E+08	10	6	5	Disturbed soil		0.857	2.47	0.22359549	0.03726592
51	2.00E+08	10	12	5	Intact soil		0.674	3.71	0.72265222	0.06022102
	2.00E+08	10	12	5	Intact soil		0.713	3.05	0.47628225	0.03969019
	2.00E+08	10	12	5	Intact soil		1.017	2.55	0.33422896	0.02785241
	2.00E+08	10	12	5	Intact soil		0.857	2.11	0.02412081	0.00201007
	2.00E+08	10	12	5	Disturbed soil		0.674	3.84	2.5195731	0.20996442
	2.00E+08	10	12	5	Disturbed soil		0.857	2.47	0.02412081	0.00201007
52	2.00E+08	0.0055	15.3	30	Disturbed soil		2.51	0.06	1.284	0.08392157
	2.00E+08	0.0055	15.3	30	Disturbed soil		2.51	0.06	1.392	0.09098039
53	3.50E+08	4.6	9.5	15	Intact soil		3.96	0.84	0.04596144	0.00483805

	3.50E+08	4.6	9.5	30	Intact soil	3.96	0.84	0.09534995	0.01003684
	3.50E+08	4.6	9.5	45	Intact soil	3.96	0.84	0.11499796	0.01210505
	3.50E+08	4.6	15.33	15	Intact soil	9.66	0.97	0.10767845	0.00702403
	3.50E+08	4.6	15.33	30	Intact soil	9.66	0.97	0.14700554	0.0095894
	3.50E+08	4.6	15.33	45	Intact soil	9.66	0.97	0.13138235	0.00857028
54	3.50E+08	1.1	2.495	15	Disturbed soil	0.95309	0.53	0.13609527	0.0545472
	3.50E+08	1.1	2.495	30	Disturbed soil	0.95309	0.53	0.08328695	0.03338154
	3.50E+08	1.1	2.495	45	Disturbed soil	0.95309	0.55	0.09138156	0.03662588
	3.50E+08	1.1	7.083	15	Intact soil	3.32901	0.53	0.10420466	0.01471194
	3.50E+08	1.1	7.083	30	Intact soil	3.32901	0.53	0.31255211	0.04412708
	3.50E+08	1.1	7.083	45	Intact soil	3.32901	0.55	0.22875674	0.03229659
	3.50E+08	1.1	4.593	15	Disturbed soil	1.488132	0.42	0.34801161	0.07577
	3.50E+08	1.1	4.593	30	Disturbed soil	1.488132	0.44	0.19022741	0.04141681
	3.50E+08	1.1	4.593	45	Disturbed soil	1.488132	0.5	0.15721499	0.03422926
	3.50E+08	1.1	6.15	15	Intact soil	2.29395	0.42	0.29124048	0.04735618
	3.50E+08	1.1	6.15	30	Intact soil	2.29395	0.44	0.30561399	0.04969333
	3.50E+08	1.1	6.15	45	Intact soil	2.29395	0.5	0.274466	0.04462862
55	4.88E+04	0.5	34	25	Intact soil	17.22	0.35	1.6286	0.0479
	6.54E+04	0.5	37	25	Intact soil	17.22	0.56	4.2513	0.1149
	7.32E+04	0.5	38	25	Intact soil	17.22	0.58	3.1882	0.0839
	5.54E+04	0.5	39	25	Intact soil	17.22	0.26	4.0287	0.1033

56	3.00E+03	1.1	0.722	20	Intact soil		0.26	0.48	0.016606	0.023
	3.00E+03	0.75	0.743	40	Intact soil		0.26	0.36	0.0392304	0.0528
57	1.00E+08	0.4	87	18.2	Disturbed soil		55.8	0.55	5.52202691	0.06347157
58	1.20E+06	7.5	5.29166667	50	Disturbed soil	180		0.6	0.11548533	0.021824
	1.20E+06	7.5	10.5833333	50	Disturbed soil	180		0.6	0.215392	0.020352
	1.20E+06	5.7	11.7083333	50	Disturbed soil	500	4.17	2.7	0.2761106	0.0235824
	1.20E+06	5.7	32.375	50	Disturbed soil	500	4.17	2.7	0.56877954	0.01756848
59	5.20E+08	30	5.96	10	Intact soil		0.84	1.45	0.55209928	0.09263411
	5.20E+08	30	4.73	10	Intact soil		1.01	1.56	0.53161894	0.11239301
60	3.50E+08	4.6	2.52	15	Disturbed soil		0.95309	0.97	0.09740951	0.03865457
	3.50E+08	4.6	2.52	30	Disturbed soil		0.95309	0.97	0.07909512	0.03138695
	3.50E+08	4.6	2.52	45	Disturbed soil		0.95309	0.97	0.06932096	0.02750832
	3.50E+08	4.6	15.42	15	Intact soil		9.66	0.97	0.06360792	0.00412503
	3.50E+08	4.6	15.42	30	Intact soil		9.66	0.97	0.14786859	0.0095894
	3.50E+08	4.6	15.42	45	Intact soil		9.66	0.97	0.12222061	0.00792611
	3.50E+08	4.6	4.68	15	Disturbed soil		1.488132	0.84	0.18090337	0.03865457
	3.50E+08	4.6	4.68	30	Disturbed soil		1.488132	0.84	0.10813096	0.02310491
	3.50E+08	4.6	4.68	45	Disturbed soil		1.488132	0.84	0.09022006	0.01927779
	3.50E+08	4.6	11.34	15	Intact soil		3.96	0.84	0.03877773	0.00341955
	3.50E+08	4.6	11.34	30	Intact soil		3.96	0.84	0.10373713	0.00914789
	3.50E+08	4.6	11.34	45	Intact soil		3.96	0.84	0.12872806	0.01135168

61	4.60E+05	20	0.09416667	20	Intact soil	0.07541667	3.535	0.0134061	0.14236561
	4.60E+05	20	0.34791667	20	Intact soil	0.27833333	3.285	0.01944498	0.05588976
	4.60E+05	20	0.38666667	20	Intact soil	0.30916667	2.99	0.01340085	0.03465736

Table S2: (a) The performance of candidate models in predicting first-order attachment coefficient (one example and averaged values from 5-fold cross validation).

Model ID	RMSE	MAE
GBM_5_AutoML_5_20230329_213518	6.876519	47.28651
XGBoost_2_AutoML_5_20230329_213518	7.20765	51.95022
GBM_grid_1_AutoML_5_20230329_213518_model_2	7.316178	53.52646
XGBoost_grid_1_AutoML_5_20230329_213518_model_3	7.476035	55.8911
XGBoost_grid_1_AutoML_5_20230329_213518_model_2	7.52997	56.70045
XGBoost_grid_1_AutoML_5_20230329_213518_model_1	7.598393	57.73557
XGBoost_3_AutoML_5_20230329_213518	7.751489	60.08558
GBM_4_AutoML_5_20230329_213518	7.849978	61.62216
XRT_1_AutoML_5_20230329_213518	7.931582	62.90999
DeepLearning_grid_2_AutoML_5_20230329_213518_model_1	7.978416	63.65512
GBM_3_AutoML_5_20230329_213518	8.228532	67.70874
GBM_2_AutoML_5_20230329_213518	8.36859	70.03329
XGBoost_1_AutoML_5_20230329_213518	8.42557	70.99022
DRF_1_AutoML_5_20230329_213518	8.724111	76.11012
DeepLearning_grid_3_AutoML_5_20230329_213518_model_1	9.753101	95.12297
GBM_grid_1_AutoML_5_20230329_213518_model_1	9.774047	95.53199
DeepLearning_grid_1_AutoML_5_20230329_213518_model_1	10.34111	106.9385
GBM_1_AutoML_5_20230329_213518	11.9224	142.1435
DeepLearning_1_AutoML_5_20230329_213518	12.66608	160.4296
GLM_1_AutoML_5_20230329_213518	12.89371	166.2478
GBM	6.842156	46.94377
XGBoost	6.802661	46.47265
DeepLearning	8.781804	77.99138
DRF	8.347773	69.77396
XRT	8.130502	66.23638
GLM	12.97116	168.6133

Table S2: (b) The performance of candidate models in predicting spatial removal rate (one example and averaged values from 5-fold cross validation).

Model ID	RMSE	MAE
GBM_5_AutoML_3_20230331_225732	0.066207	0.004383
XGBoost_3_AutoML_3_20230331_225732	0.06785	0.004604
XGBoost_grid_1_AutoML_3_20230331_225732_model_2	0.073635	0.005422
XGBoost_grid_1_AutoML_3_20230331_225732_model_3	0.074827	0.005599
XGBoost_2_AutoML_3_20230331_225732	0.076888	0.005912
DRF_1_AutoML_3_20230331_225732	0.08459	0.007156
GBM_grid_1_AutoML_3_20230331_225732_model_2	0.085605	0.007328
GBM_2_AutoML_3_20230331_225732	0.087612	0.007676
GBM_3_AutoML_3_20230331_225732	0.088714	0.00787
GBM_4_AutoML_3_20230331_225732	0.090402	0.008172
XGBoost_1_AutoML_3_20230331_225732	0.092268	0.008513
XRT_1_AutoML_3_20230331_225732	0.101373	0.010276
GBM_grid_1_AutoML_3_20230331_225732_model_1	0.113753	0.01294
XGBoost_grid_1_AutoML_3_20230331_225732_model_1	0.115458	0.01333
DeepLearning_grid_2_AutoML_3_20230331_225732_model_1	0.126975	0.016123
DeepLearning_grid_1_AutoML_3_20230331_225732_model_1	0.131267	0.017231
DeepLearning_grid_3_AutoML_3_20230331_225732_model_1	0.142589	0.020332
GBM_1_AutoML_3_20230331_225732	0.146386	0.021429
GLM_1_AutoML_3_20230331_225732	0.147182	0.021663
DeepLearning_1_AutoML_3_20230331_225732	0.149172	0.022252
GBM	0.067772	0.005586
XGBoost	0.067376	0.005509
DeepLearning	0.098221	0.011626
DRF	0.080607	0.007842
XRT	0.083007	0.008277
GLM	0.116385	0.016353

Code in R used for training H2O AutoML.

```
# Start H2O AutoML
library(h2o)
h2o.init()
# Import training datasets and testing datasets
train <- h2o.importFile("G:/Chen Fengxian 2022.4.27/ML/EcoliML/AML/FAC-train.csv")
test <- h2o.importFile("G:/Chen Fengxian 2022.4.27/ML/EcoliML/AML/FAC-test.csv")
# Name the input variables and target variables
y <- "AC"
x <- setdiff(names(train), y)
print(x)
print(y)
# Define the porous medium type as categorical variable
train["PM"] <- as.factor(train["PM"])
test["PM"] <- as.factor(test["PM"])
# Model training
aml <- h2o.automl(x = x, y = y,
                  training_frame = train,
                  max_models = 20,
                  seed = 1)
# Print the leaderboard according to the model performance
lb <- aml@leaderboard
print(lb, n = nrow(lb))
#Print the predicted results
pred <- h2o.predict(aml@leader, test)
print(pred, n = nrow(pred))
#Print the variable importance of the best model
varimp <- h2o.varimp(aml)
print (varimp)
#Get the best model based on its ID
```

```r
gbm5 <- h2o.getModel ("GBM_5_AutoML_1_20221215_164033")
#Plot the SHAP summary plot of the best tree-based model
shap_summary_plot <- h2o.shap_summary_plot (gbm5, test)
print(shap_summary_plot)
```

Reference

1. Wang, S., Zhang, M., He, L., Li, M., Zhang, X., Liu, F. and Tong, M., 2022. Bacterial Capture and Inactivation in Sand Filtration Systems With Addition of Zero-valent Iron As Permeable Layer Under Both Slow and Fast Filtration Conditions. *Journal of Hazardous Materials*, p.129122.
2. He, L., Li, M., Wu, D., Guo, J., Zhang, M. and Tong, M., 2022. Freeze-Thaw Cycles Induce Diverse Bacteria Release Behaviors from Quartz Sand Columns with Different Water Saturations. *Water Research*, p.118683.
3. Yang, L., Kang, J., Chen, X., Ripp, S.A., Johnson, W.P. and Zhuang, J., 2021. Real-time bioluminescent imaging of spatiotemporal variation of microbial retention during transport through porous media under variably saturated flow conditions. *Journal of Hydrology*, *601*, p.126603.
4. Park, S.J., Kim, S.B. and Kim, K.W., 2010. Analysis of bacterial cell properties and transport in porous media. *Journal of Environmental Science and Health Part A*, *45*(6), pp.682-691.
5. Sasidharan, S., Bradford, S.A., Torkzaban, S., Ye, X., Vanderzalm, J., Du, X. and Page, D., 2017. Unraveling the complexities of the velocity dependency of E. coli retention and release parameters in saturated porous media. *Science of the Total Environment*, *603*, pp.406-415.
6. Wu, D., He, L., Sun, R., Tong, M. and Kim, H., 2017. Influence of Bisphenol A on the transport and deposition behaviors of bacteria in quartz sand. *Water research*, *121*, pp.1-10.
7. Madumathi, G., Philip, L. and Bhallamudi, S.M., 2017. Transport of E. coli in saturated and unsaturated porous media: effect of physiological state and substrate availability. *Sādhanā*, *42*, pp.1007-1024.
8. He, L., Wu, D., Rong, H., Li, M., Tong, M. and Kim, H., 2018. Influence of nano-and microplastic particles on the transport and deposition behaviors of bacteria in quartz sand. *Environmental Science & Technology*, *52*(20), pp.11555-11563.
9. Hong, Z.N., Jiang, J., Li, J.Y., Xu, R.K. and Yan, J., 2019. Adhesion mediated transport of bacterial pathogens in saturated sands coated by phyllosilicates and Al-oxides. *Colloids and Surfaces B: Biointerfaces*, *181*, pp.215-225.
10. Bai, H., Chen, J., Gao, W., Yang, B., Yan, Y., Liu, W., Wang, G. and Lamy, E., 2021. Transport and retention of bacteria through a filtration system consisting of sands and geotextiles. *Colloids and Surfaces B: Biointerfaces*, *208*, p.112114.
11. Du, M., Wang, L., Ebrahimi, A., Chen, G., Shu, S., Zhu, K., Shen, C., Li, B. and Wang, G., 2021. Extracellular polymeric substances induced cell-surface interactions facilitate bacteria transport in saturated porous media. *Ecotoxicology and Environmental Safety*, *218*, p.112291.
12. Gao, M., Peng, H. and Xiao, L., 2021. The influence of microplastics for the transportation of E. coli using column model. *Science of The Total Environment*, *786*, p.147487.
13. Zhang, M., He, L., Jin, X., Bai, F., Tong, M. and Ni, J., 2021. Flagella and their properties affect the transport and deposition behaviors of Escherichia coli in quartz sand. *Environmental Science & Technology*, *55*(8), pp.4964-4973.
14. Zhang, D. and Prigiobbe, V., 2022. Measuring and modeling the influence of salinity change on the transport behaviour of Escherichia coli through quartz sand. *Journal of Contaminant Hydrology*, *248*, p.104016.
15. Dong, Z., Yang, H., Wu, D., Ni, J., Kim, H. and Tong, M., 2014. Influence of silicate on the transport of bacteria in quartz sand and iron mineral-coated sand. *Colloids and Surfaces B: Biointerfaces*, *123*, pp.995-1002.
16. Li, J., Zhao, X., Tian, X., Li, J., Sjollema, J. and Wang, A., 2015. Retention in treated wastewater affects survival and deposition of Staphylococcus aureus and Escherichia

coli in sand columns. *Applied and environmental microbiology*, *81*(6), pp.2199-2205.

17. Bai, H., Cochet, N., Pauss, A. and Lamy, E., 2016. Bacteria cell properties and grain size impact on bacteria transport and deposition in porous media. *Colloids and Surfaces B: Biointerfaces*, *139*, pp.148-155.
18. Shen, X., Han, P., Yang, H., Kim, H. and Tong, M., 2013. Influence of sulfate on the transport of bacteria in quartz sand. *Colloids and Surfaces B: Biointerfaces*, *110*, pp.443-449.
19. Yang, H., Kim, H. and Tong, M., 2012. Influence of humic acid on the transport behavior of bacteria in quartz sand. *Colloids and Surfaces B: Biointerfaces*, *91*, pp.122-129.
20. Kim, H.N. and Walker, S.L., 2009. Escherichia coli transport in porous media: Influence of cell strain, solution chemistry, and temperature. *Colloids and Surfaces B: Biointerfaces*, *71*(1), pp.160-167.
21. Bradford, S.A., Simunek, J. and Walker, S.L., 2006. Transport and straining of E. coli O157: H7 in saturated porous media. *Water Resources Research*, *42*(12).
22. Liu, J., Ford, R.M. and Smith, J.A., 2011. Idling time of motile bacteria contributes to retardation and dispersion in sand porous medium. *Environmental science & technology*, *45*(9), pp.3945-3951.
23. Sasidharan, S., Torkzaban, S., Bradford, S.A., Kookana, R., Page, D. and Cook, P.G., 2016. Transport and retention of bacteria and viruses in biochar-amended sand. *Science of the Total Environment*, *548*, pp.100-109.
24. Chung, J.W., Foppen, J.W., Izquierdo, M. and Lens, P.N., 2014. Removal of Escherichia coli from saturated sand columns supplemented with hydrochar produced from maize. *Journal of environmental quality*, *43*(6), pp.2096-2103.
25. Walczak, J.J., Wang, L., Feriancikova, L., Li, J. and Xu, S., 2012. Influence of desiccation on the transport of Escherichia coli through saturated sand packs. *Water, Air, & Soil Pollution*, *223*, pp.1353-1362.
26. Syngouna, V.I. and Chrysikopoulos, C.V., 2011. Transport of biocolloids in water saturated columns packed with sand: Effect of grain size and pore water velocity. *Journal of Contaminant Hydrology*, *126*(3-4), pp.301-314.
27. Walczak, J.J., Wang, L., Bardy, S.L., Feriancikova, L., Li, J. and Xu, S., 2012. The effects of starvation on the transport of Escherichia coli in saturated porous media are dependent on pH and ionic strength. *Colloids and surfaces B: Biointerfaces*, *90*, pp.129-136.
28. Walczak, J.J., Bardy, S.L., Feriancikova, L. and Xu, S., 2011. Influence of tetracycline resistance on the transport of manure-derived Escherichia coli in saturated porous media. *Water Research*, *45*(4), pp.1681-1690.
29. Kim, S.B., Park, S.J., Lee, C.G. and Kim, H.C., 2008. Transport and retention of Escherichia coli in a mixture of quartz, Al‐coated and Fe‐coated sands. *Hydrological Processes: An International Journal*, *22*(18), pp.3856-3863.
30. Torkzaban, S., Tazehkand, S.S., Walker, S.L. and Bradford, S.A., 2008. Transport and fate of bacteria in porous media: Coupled effects of chemical conditions and pore space geometry. *Water Resources Research*, *44*(4).
31. Jiang, G., Noonan, M.J., Buchan, G.D. and Smith, N., 2007. Transport of Escherichia coli through variably saturated sand columns and modeling approaches. *Journal of Contaminant Hydrology*, *93*(1-4), pp.2-20.
32. Bolster, C.H., Walker, S.L. and Cook, K.L., 2006. Comparison of Escherichia coli and Campylobacter jejuni transport in saturated porous media. *Journal of environmental quality*, *35*(4), pp.1018-1025.

33. Redman, J.A., Walker, S.L. and Elimelech, M., 2004. Bacterial adhesion and transport in porous media: Role of the secondary energy minimum. *Environmental science & technology*, *38*(6), pp.1777-1785.

34. Zhang, X., Chen, F., Yang, L., Qin, F. and Zhuang, J., 2022. Quantifying bacterial concentration in water and sand media during flow-through experiments using a non-invasive, real-time, and efficient method. *Frontiers in Microbiology*, *13*.

35. Tazehkand, S.S., Torkzaban, S., Bradford, S.A. and Walker, S.L., 2008. Cell preparation methods influence Escherichia coli D21g surface chemistry and transport in saturated sand. *Journal of environmental quality*, *37*(6), pp.2108-2115.

36. Foppen, J.W., Van Herwerden, M. and Schijven, J., 2007. Measuring and modelling straining of Escherichia coli in saturated porous media. *Journal of contaminant hydrology*, *93*(1-4), pp.236-254.

37. Wang, Y., Bradford, S.A. and Šimůnek, J., 2013. Transport and fate of microorganisms in soils with preferential flow under different solution chemistry conditions. *Water resources research*, *49*(5), pp.2424-2436.

38. Eisfeld, C., Schijven, J.F., van der Wolf, J.M., Medema, G., Kruisdijk, E. and van Breukelen, B.M., 2022. Removal of bacterial plant pathogens in columns filled with quartz and natural sediments under anoxic and oxygenated conditions. *Water Research*, *220*, p.118724.

39. Chalotra, A., Ratha, D., Babbar, R. and Baranwal, M., 2022. A study on transport of Escherichia coli through saturated porous medium. *International Journal of Environmental Science and Technology*, pp.1-16.

40. Lutterodt, G., Foppen, J.W.A. and Uhlenbrook, S., 2012. Transport of Escherichia coli strains isolated from natural spring water. *Journal of contaminant hydrology*, *140*, pp.12-20.

41. Lutterodt, G., Foppen, J.W.A., Maksoud, A. and Uhlenbrook, S., 2011. Transport of Escherichia coli in 25 m quartz sand columns. *Journal of contaminant hydrology*, *119*(1-4), pp.80-88.

42. Powelson, D.K. and Mills, A.L., 2001. Transport of Escherichia coli in sand columns with constant and changing water contents. *Journal of environmental quality*, *30*(1), pp.238-245.

43. Wang, L., Xu, S. and Li, J., 2011. Effects of phosphate on the transport of Escherichia coli O157: H7 in saturated quartz sand. *Environmental science & technology*, *45*(22), pp.9566-9573.

44. Johanson, J.J., Feriancikova, L., Banerjee, A., Saffarini, D.A., Wang, L., Li, J., Grundl, T.J. and Xu, S., 2014. Comparison of the transport of Bacteroides fragilis and Escherichia coli within saturated sand packs. *Colloids and Surfaces B: Biointerfaces*, *123*, pp.439-445.

45. Bai, H., Cochet, N., Drelich, A., Pauss, A. and Lamy, E., 2016. Comparison of transport between two bacteria in saturated porous media with distinct pore size distribution. *Rsc Advances*, *6*(18), pp.14602-14614.

46. Yao, S.Y., Yuan, X.M., Yang, X.Y. and Deng, S.H., 2016. Effects of particle size and pore water velocity on transport of Escherichia coli in saturated porous media. *Journal of Agro-Environment Science*, *35*(02), pp.353-357.

47. Haznedaroglu, B.Z., Bolster, C.H. and Walker, S.L., 2008. The role of starvation on Escherichia coli adhesion and transport in saturated porous media. *Water research*, *42*(6-7), pp.1547-1554.

48. Abu-Lail, N.I. and Camesano, T.A., 2003. Role of lipopolysaccharides in the adhesion, retention, and transport of Escherichia coli JM109. *Environmental science &*

technology, *37*(10), pp.2173-2183.

49. Won, J., Kim, J.W., Kang, S. and Choi, H., 2007. Transport and adhesion of Escherichia coli JM109 in soil aquifer treatment (SAT): One-dimensional column study. *Environmental monitoring and assessment*, *129*, pp.9-18.

50. Chen, J., Yang, L., Chen, X., Ripp, S., Radosevich, M. and Zhuang, J., 2021. Bacterial mobility facilitated by soil depth and intact structure. *Soil and Tillage Research*, *209*, p.104911.

51. Chen, J., Yang, L., Chen, X., Ripp, S. and Zhuang, J., 2022. Coupled effects of pore water velocity and soil heterogeneity on bacterial transport: intact vs. repacked soils. *Frontiers in Microbiology*, *13*.

52. Park, S.J., Kim, S.B. and Kim, K.W., 2010. Analysis of bacterial cell properties and transport in porous media. *Journal of Environmental Science and Health Part A*, *45*(6), pp.682-691.

53. Gharabaghi, B., Safadoust, A., Mahboubi, A.A., Mosaddeghi, M.R., Unc, A., Ahrens, B. and Sayyad, G., 2015. Temperature effect on the transport of bromide and E. coli NAR in saturated soils. *Journal of Hydrology*, *522*, pp.418-427.

54. Safadoust, A., Mahboubi, A.A., Mosaddeghi, M.R., Gharabaghi, B., Voroney, P., Unc, A. and Khodakaramian, G., 2012. Significance of physical weathering of two-texturally different soils for the saturated transport of Escherichia coli and bromide. *Journal of environmental management*, *107*, pp.147-158.

55. Sepehrnia, N., Mahboubi, A.A., Mosaddeghi, M.R., Sinejani, A.S. and Khodakaramian, G., 2014. Escherichia coli transport through intact gypsiferous and calcareous soils during saturated and unsaturated flows. *Geoderma*, *217*, pp.83-89.

56. Mosaddeghi, M.R., Sinegani, A.S., Farhangi, M.B., Mahboubi, A.A. and Unc, A., 2010. Saturated and unsaturated transport of cow manure-borne Escherichia coli through in situ clay loam lysimeters. *Agriculture, ecosystems & environment*, *137*(1-2), pp.163-171.

57. Sepehrnia, N., Bachmann, J., Hajabbasi, M.A., Rezanezhad, F., Lichner, L., Hallett, P.D. and Coyne, M., 2019. Transport, retention, and release of Escherichia coli and Rhodococcus erythropolis through dry natural soils as affected by water repellency. *Science of The Total Environment*, *694*, p.133666.

58. Hijnen, W.A., Brouwer-Hanzens, A.J., Charles, K.J. and Medema, G.J., 2005. Transport of MS2 phage, Escherichia coli, Clostridium perfringens, Cryptosporidium parvum, and Giardia intestinalis in a gravel and a sandy soil. *Environmental science & technology*, *39*(20), pp.7860-7868.

59. Firouzi, A.F., Homaee, M., Klumpp, E., Kasteel, R. and Tappe, W., 2015. Bacteria transport and retention in intact calcareous soil columns under saturated flow conditions. *Journal of Hydrology and Hydromechanics*, *63*(2), pp.102-109.

60. Safadoust, A., Mahboubi, A.A., Gharabaghi, B., Mosaddeghi, M.R., Voroney, P., Unc, A. and Sayyad, G., 2011. Bacterial filtration rates in repacked and weathered soil columns. *Geoderma*, *167*, pp.204-213.

61. Guber, A.K., Shelton, D.R. and Pachepsky, Y.A., 2005. Transport and retention of manure-borne coliforms in soil. *Vadose Zone Journal*, *4*(3), pp.828-837.
